# Sensing soluble uric acid by Naip1-Nlrp3 platform

**DOI:** 10.1038/s41419-021-03445-w

**Published:** 2021-02-05

**Authors:** Tarcio Teodoro Braga, Mariana Rodrigues Davanso, Davi Mendes, Tiago Antonio de Souza, Anderson Fernandes de Brito, Mario Costa Cruz, Meire Ioshie Hiyane, Dhemerson Souza de Lima, Vinicius Nunes, Juliana de Fátima Giarola, Denio Emanuel Pires Souto, Tomasz Próchnicki, Mario Lauterbach, Stellee Marcela Petris Biscaia, Rilton Alves de Freitas, Rui Curi, Alessandra Pontillo, Eicke Latz, Niels Olsen Saraiva Camara

**Affiliations:** 1 Department of Basic Pathology, Federal University of Parana, Curitiba, PR Brazil; 2 Department of Immunology, Institute of Biomedical Sciences IV, University of São Paulo, São Paulo, SP Brazil; 3 Institute of Innate Immunity, University Hospitals Bonn, Bonn, Germany; 4 Department of Physiology and Biophysics, Institute of Biomedical Sciences I, University of Sao Paulo, São Paulo, SP Brazil; 5 Department of Microbiology, Institute of Biomedical Sciences II, University of São Paulo, São Paulo, SP Brazil; 6 Department of Life Sciences, Imperial College London, London, SW7 2AZ UK; 7 Institute of Chemistry, University of Campinas, Campinas, SP Brazil; 8 Department of Chemistry, Federal University of Parana, Curitiba, PR Brazil; 9 Department of Cellular Biology, Federal University of Parana, Curitiba, PR Brazil; 10 Interdisciplinary Post-Graduate Program in Health Sciences, Cruzeiro do Sul University, São Paulo, Brazil; 11 Division of Infectious Diseases and Immunology, Department of Medicine, University of Massachusetts Medical School, Worcester, MA 01655 USA; 12 Centre for Molecular Inflammation Research (CEMIR), Norwegian University of Science and Technology, 7491 Trondheim, Norway; 13 Nephrology Division, Federal University of São Paulo, São Paulo, SP Brazil; 14 Renal Physiopathology Laboratory, Faculty of Medicine, University of São Paulo, São Paulo, SP Brazil

**Keywords:** NOD-like receptors, Endocrine system and metabolic diseases

## Abstract

Uric acid (UA), a product of purine nucleotide degradation able to initiate an immune response, represents a breakpoint in the evolutionary history of humans, when uricase, the enzyme required for UA cleavage, was lost. Despite being inert in human cells, UA in its soluble form (sUA) can increase the level of interleukin-1β (IL-1β) in murine macrophages. We, therefore, hypothesized that the recognition of sUA is achieved by the Naip1-Nlrp3 inflammasome platform. Through structural modelling predictions and transcriptome and functional analyses, we found that murine Naip1 expression in human macrophages induces IL-1β expression, fatty acid production and an inflammation-related response upon sUA stimulation, a process reversed by the pharmacological and genetic inhibition of Nlrp3. Moreover, molecular interaction experiments showed that Naip1 directly recognizes sUA. Accordingly, Naip may be the sUA receptor lost through the human evolutionary process, and a better understanding of its recognition may lead to novel anti-hyperuricaemia therapies.

## Introduction

Host responses against harmful signals are basic physiological reactions of all living organisms. Innate immunity pattern-recognition receptors (PRRs) were first described as recognizing conserved structural components of microorganisms^1^. The discovery of Toll-like receptors^2^ led scientists to understand how the immune system responds to nonself antigens in the context of an infection^3,4^, in contrast with the previous model, in which the immune system reacted to all nonself antigens while being tolerant to self-antigens^5,6^. Based on Polly Matzinger’s study stating that “the immune system is more concerned with entities that do damage than with those that are foreign”^6^, several damage-associated molecular patterns (DAMPs) have been described. Indeed, receptors for endogenous and exogenous signals may have evolved simultaneously because vertebrates and pathogens have shared eons of evolutionary time and space^6^. Perhaps, PRRs have not evolved to bind to pathogens at all; in contrast, perhaps, the pathogens evolved to attach to PRRs and thus enhance their own survival^7^, a hypothesis that would explain a puzzling feature of PRRs: each one can attach to many different kinds of molecules.

Among several DAMPs, uric acid (UA), the product of purine catabolism, released mainly from dying cells and ischaemic tissues, is considered a major alarmin, especially when it is present at elevated levels and crystallized, also known as monosodium urate (MSU)^8^. In rodents, MSU activates the immune system^9,10^, acts as a pro-oxidant molecule, stimulates chemotaxis and activates the nuclear factor-κB and MAPK pathways^11^. Moreover, MSU induces the release of interleukin-1β (IL-1β) through the activation of inflammasome-dependent caspases^10,12,13^. The inflammasome is a cytosolic complex activated when a nucleotide-binding domain (NBD/NACHT) and leucine-rich repeat (LRR)-containing receptor (NLR) senses PAMPs or DAMPs^14^. NLRs belong to a superfamily of innate immune proteins with a very conserved structure throughout the phylogeny, from plants to mammals. In distinct phylogenetic groups, the number of receptors and paralogous genes differs, possibly as a consequence of host/pathogen and/or environmental coevolution^15^. To date, it has been demonstrated that both soluble UA (sUA)^16^ and MSU can induce Nlrp3 (an NLR containing a pyrin domain as its N-terminal domain) oligomerization in mice, while the deposition of MSU activates Nlrp3 inflammasome in humans, in the context of gout ^17^.

Great apes have higher levels of UA in serum (3.02–6.72 mg/dL, corresponding to 180–400 μΜ) than other animals (18–40 μΜ) and UA crystallization occurs, in humans, when the level reaches 6.8 mg/dL in plasma^18^. This observation is compatible with the absence of uricase (or urate oxidase) activity, the enzyme involved in purine catabolism converting UA into allantoin^19,20^. The loss of uricase at the divergence between great apes and other mammals may be related to a survival advantage, as previously hypothesized, due to the UA characteristics as a molecule critical for saving energy^21^; however, this supposition raises a tricky question about the role of a mammalian sensor of UA. We hypothesize that, along with uricase loss and a consequent elevation in UA serum levels, humans have lost the sensors to recognize “high” levels of sUA.

Among NLRs able to induce inflammasome activation in mice and humans, the NLRB subfamily (an NLR containing a baculovirus inhibitor of apoptosis protein repeat domain as its N-terminal domain) attracted our attention. In mice, there are six paralogous genes, namely, *Naip*1–6, and four functional receptors (Naip1, 2, 5 and 6), while in humans, only one orthologous gene, *Naip*, has been found^22^. Despite the differences in the amino acid content among Naip proteins, both mice and human receptors have been described to play role in the host defence against pathogens^23,24^. Murine (m) Naip1 and Naip2 are critical for the detection of needle and rod proteins, respectively^25–27^. Naip5 is crucial for the cytosolic recognition of flagellin^28^, the major protein component of the bacterial flagellum. Similar to Naip5, human Naip (hNaip) can bind bacterial flagellin^29^ and activate the Nlrc4 inflammasome. In this scenario, Naip acts as a ligand sensor, and Nlrc4 is critical for inflammasome assembling and inflammasome-dependent cell death, known as pyroptosis^30^. Considering the differences in NLRB orthologous genes among different species and the lack of endogenous ligands found for NLRB to date, in this study, we performed transcriptome- and proteome-wide analyses in addition to interaction investigations and structural modelling predictions to study the sensing of sUA by mNaip1. In addition to demonstrating that mNaip1 expression leads to the activation in human cells upon sUA stimulation, we found that mNaip1 directly recognizes sUA. We then hypothesized that Naip may be the lost receptor for UA and, in particular, for sUA.

## Materials and methods

### Soluble UA preparation

The medium was prewarmed (37 °C), UA (Ultrapure, Sigma; 200 μΜ) was added, and the medium was sterilized with 0.20 μm filters. Crystals were not detectable under these conditions (polarizing microscopy), nor did they develop during cell incubation.

### Reagents

Ultrapure LPS (lipopolysaccharide) was obtained from InvivoGen, and nigericin was obtained from Invitrogen. DRAQ5 was purchased from eBioscience. Anti-GFP monoclonal antibody (GF28R) was purchased from Thermo Fisher Scientific. CRID3 was purchased from R&D Systems, and allantoin, urea and palmitate were purchased from Sigma-Aldrich. TOFA (5-(tetradecyloxy)-2-furoic acid) (CAS 54857-86-2) was purchased from Abcam. C75 (CAS 191282-48-1) was also obtained from Abcam. Cerulenin (17397-89-6) was purchased from Sigma-Aldrich, and BMS303141 (CAS 943962-47-8) was purchased from Cayman Chemical. UK5099 CAS 56396-35-1 was obtained from Merck Millipore. Ethanolamine (EA), 11-mercaptoundecanoic acid (11-MUA), *N*-(3 dimethylamino-propyl)-*N*-ethylcarbodiimide hydrochloride (EDC), and *N*-hydroxysuccinimide (NHS) were obtained from Sigma-Aldrich Chemical (St. Louis, MO, USA).

### Macrophages obtainment

Primary murine macrophages were generated from bone marrow. Briefly, bone marrow-derived cells were filtered through sterile polystyrene syringes (70–100 µm) and divided among 10 Petri dishes (100 × 20 mm^2^; BD, Franklin Lakes, USA) supplemented with murine macrophage CSF (20 ng/mL; R&D Systems, Minneapolis, MN, USA) diluted in high-glucose Dulbecco’s modified Eagle’s medium (Invitrogen, USA) in the presence of 5% foetal bovine serum for 7 days. All procedures with mice were approved by the local ethics committees at the University of São Paulo (Document 45/2009). All experiments were performed in accordance with relevant guidelines and regulations.

Human and rhesus macaque (*Macaca mulatta*) macrophages were generated from circulating monocytes. A centrifugation gradient was used to obtain human and monkey peripheral blood mononuclear cells in Ficoll-Paque (Dominique Dutscher). Subsequently, the peripheral blood mononuclear cells were centrifuged in 51% phosphate-buffered saline-diluted Percoll (GE Healthcare Life Sciences), and the monocytes (>70% purity) were allowed to adhere to six-well plates for 4 h. After removal of nonadherent cells by washing the plates, the monocytes were differentiated into macrophages in RPMI medium (Gibco, Grand Island, NY, USA) supplemented with 10% foetal calf serum (Gibco) plus antibiotics and antimycotics (100 U/mL penicillin, 100 g/mL streptomycin and 25 g/mL amphotericin; Gibco) in the presence of human macrophage CSF (25 ng/mL; R&D Systems, Minneapolis, MN, USA) for 5 days. All procedures with rhesus macaques were approved by the Ethics Committee on Animal Use of the Butantan Institute (CEUAIB) (number 9376040717). Leucocyte concentrates were obtained after plasmapheresis at the Blood Bank Service of the “Hospital das Clinicas” in Sao Paulo (SP, Brazil), and they were used for peripheral blood monocyte isolation. All volunteers signed informed consent in compliance with the respective Institutional Ethics Committee. We performed power and sample size calculations to determine how large the sample needed to be to obtain 90% power when comparing a categorical variable among groups. Experiments were performed in duplicate or triplicate, and at least two independent tests were performed for each assay. The data are described in terms of the means and s.e.m. unless specified in the figure legend.

### Viral transduction

Plasmids for GFP-tagged mNaip1 (#60200), Naip5 (#60205), Naip6 (#60202), Nlrc4 (#60199) and empty vector (#60206) were purchased from Addgene (Watertown, MA, USA). Transformed bacteria with the GFP-tagged mNaip2 (#60201) plasmid were not able to grow. The plasmid was additionally modified by adding a “self-cleaving” 2A (T2A) sequence between Naip1 and the colour-tagged sequences with standard cloning techniques. Briefly, THP1 cells (ATCC, TIB202) were retrovirally transduced with constructs for the indicated plasmids. After retroviral transduction, the cells were cytometrically sorted with cells with similar levels of GFP or turquoise expression. Nlrp3-deficient (Nlrp3^−/−)^ macrophages have been previously described^31^, and Nlrp3 deletion from THP1 cells was performed according to the procedure described by Schmid-Burgk et al.^32^.

### Cytokine profile

Cell lysates were maintained in RIPA buffer with protease inhibitors at −80 °C until dosing. IL-1β protein was measured using IL-1β (R&D Systems, Minneapolis, MN, USA) according to the manufacturer’s instructions.

### RNA extraction, library construction and sequencing

Total RNA was extracted from GFP-sorted THP1 cells containing the lentiviral vector NAIP1 (*n* = 6) or the empty vector GFP^+^ control cells (*n* = 6) using TRIzol reagent according to the manufacturer’s instructions (Thermo Fisher Scientific, Waltham, MA, USA). The integrity of the total RNA was checked using a Bioanalyzer 2100 (Agilent Technologies, CA, USA) with an RNA Nano 6000 kit. RNA purity and quantity were measured using a NanoDrop 1000 spectrophotometer and Qubit RNA HS fluorescence kit (Thermo Fisher), respectively. The total RNA concentration of each sample (*n* = 12) was adjusted to 1 µg and used for poly(A) messenger RNA (mRNA) enrichment and strand-specific RNA-Seq (RNA sequencing) library preparation with a TruSeq Stranded mRNA kit (Illumina, San Diego, CA, USA). Quality control of the prepared libraries was performed using an Agilent Bioanalyzer DNA 1000 kit and Qubit DNA HS fluorescence kit. The libraries were pooled and then sequenced at CEFAP-USP (Sao Paulo, Brazil) on an Illumina NextSeq platform in 75-bp paired-end mode using a high output kit.

### RNA-Seq data analysis

Before read mapping, clean reads were selected after preprocessing with Trimmomatic^33^ to remove adapter and poly(N) sequences. After cleaning, the quality of reads was checked by the FastQC tool and then aligned to the human genome (GRCh38/hg38) using the HISAT2 aligner (V2-2.0.0)^34^ considering strandness. The overall mapping quality and uniformity of the read coverage on exons were checked by the RSeQC tool to ensure good RNA integrity and reproducible RNA-Seq. The StringTie (v.1.3.4)^35^ and Ballgown^36^ algorithms were applied to identify significantly differentially expressed genes (*q* value <0.05) based on the “new Tuxedo” package^37^. Gene set enrichment analyses were performed using the Enrichr tool and GAGE package to identify upregulated (fold change (FC) > 1 and *q* value < 0.05) and downregulated genes (FC < 1 and *q* value < 0.05). High-throughput sequence data were uploaded as supplemental material.

### Proteomics analysis

Protein solutions were quantified by fluorometry using a Qubit^®^ protein assay kit. From these solutions, an equivalent aliquot of 50 μg of protein was transferred to 0.5 mL tubes and dried. The protein pellets were suspended in 6 M urea in aqueous solution (25 μL). The same volume of reducing reagent plus 10 mM dithiothreitol was added, and the samples were reduced for 60 min at room temperature. Subsequently, 50 μL of an alkylation solution, 100 mM iodoacetamide, was added, and the samples were alkylated for another 60 min at 54 °C in the dark. Next, 1 M dithiothreitol (1 μL) was added and allowed to react with the remaining iodoacetamide. Finally, 100 μL of ice-cold trypsin solution at a 1:50 (trypsin:protein) ratio was added to the samples, followed by incubation for 16 h at 37 °C. Following digestion, the reaction was stopped by adding 10% formic acid (5 μL). The samples were then desalted using ZipTips® and maintained at −20 °C until analysis.

Peptides were analysed by online nanoflow LC-MS (liquid chromatography-mass spectrometry) in an EASY-nLC II system (Thermo Scientific) connected to an LTQ-Orbitrap Velos instrument (Thermo Scientific) and a Proxeon nanoelectrospray ion source. The peptides were separated on an analytical EASY column (10 cm, ID 75 μm, 3 μm, C18; Thermo Scientific) previously trapped in a precolumn EASY column (2 cm, ID100 μm, 5 μm, C18; Thermo Scientific). Tryptic digested peptides were separated using a 60 min linear gradient of 0–60% buffer B (acetonitrile in 0.1% formic acid) at a 300 nL/min flow rate. The LTQ-Orbitrap Velos mass spectrometer was operated in positive ion mode using DDA (data-dependent acquisition) mode. Full mass spectrometric (MS) scans were performed with 60,000 resolution, and the *m*/*z* range for the MS scans was 400–1200. The minimum signal threshold was 15,000 counts, and for dynamic exclusion, it was considered a one repeat count with a duration of 30 s. To discriminate the charge state of the peptides, charge state screening was enabled, and ions with an unassigned charge state or singly charged ions were rejected.

The MS/MS spectra from each LC-MS/MS run were compared against five different databases with two distinct search engines, an in-house program and Proteome Discoverer 1.4 software (Thermo, USA). The search criteria were as follows: full tryptic specificity was required, two missed cleavages were allowed, carbamidomethylation (C) was set as the fixed modification, oxidation (M) was set as the variable modification, precursor ion mass tolerance was set at 10 p.p.m. for all the MS spectra acquired with the Orbitrap mass analyser, and the fragment ion mass tolerance was set at 0.6 Da for all the MS2 spectra acquired. All covariates were log-transformed before statistical analysis was performed. All analyses were performed using STRING software and UniProt for protein–protein interactions, identification, and statistics. *P* ≤ 0.05 was considered significant.

### Oxygen consumption rates

An hour before the oxygen consumption measurements, the cell medium was replaced by assay medium (2 mM glucose, 0.8 mM Mg^2+^, 1.8 mM Ca^2+^, 143 mM NaCl, 5.4 mM KCl, 0.91 mM NaH_2_PO_4_ and 15 mg/mL Phenol red) and incubated for 60 min at 37 °C (no CO_2_) before loading into a Seahorse Bioscience XF96 extracellular analyser. During the 60-min period, the ports of the cartridge containing the oxygen probes were loaded with the compounds to be injected during the assay (75 μL/port), and the cartridge was calibrated. Basal respiration was recorded for 30 min at 4-min intervals until system stabilization. CCCP (carbonyl cyanide *p*-trifluoromethoxyphenylhydrazone) was used at a final concentration of 5 mM and injected with sodium pyruvate (Sigma) at a final concentration of 5 mM. Oligomycin and antimycin A were used at final concentrations of 1 and 10 μg/mL, respectively. Rotenone was used at a concentration of 1 μM. All respiratory modulators were used at ideal concentrations titrated during preliminary experiments (not shown). A typical oxygen consumption ratio (OCR) chart is displayed, where OCR represents the percentage of basal respiration.

### Western blotting

Proteins derived from cell lysates were separated on sodium dodecyl sulfate-polyacrylamide gel electrophoresis gels (4–12%) (Novex, Invitrogen) with 2-(N-morpholino) ethanesulfonic acid buffer (Novex, Invitrogen) at 150 V from 60 to 90 min. The proteins were transferred onto polyvinylidene difluoride membranes (Millipore, Temecula, CA, USA), which had been pretreated for 1–2 min with methanol, at 32 V for 90 min in buffer containing 10% Tris-glycine and 15% methanol. The membranes were blocked with 3% bovine serum albumin (BSA) containing Tris buffer in saline solution (TBS) for 60 min. After blocking, the membranes were incubated with specific primary antibodies in TBS containing 3% BSA and 0.1% Tween-20 overnight. The following primary antibodies were used: goat anti-mouse Naip1 (sc-11067, 1:200; Santa Cruz Biotechnology, Santa Cruz, CA, USA), rabbit anti-mouse NLRP3 (mAb #15101, 1:1000; Cell Signalling Technology, Danvers, MA, USA), anti-β-actin mouse (1:1000, Li-COR Bioscience, Lincoln, NE, USA) and anti-IL-1β from an R&D ELISA kit as a detection antibody (1:1000). The membranes were washed and incubated with secondary antibodies (IRDye 800CW or IrDye 680RD, LI-COR Biosciences, 1:20,000) for 60 min. After washing, the stained cells were visualized on an Odyssey imaging system. The staining intensity was quantified using the Fiji/ImageJ software.

### Confocal imaging

Confocal laser scanning microscopy was performed with a Leica TCS SP5 SMD confocal system (Leica Microsystems). The images were captured using a single z step, and the emitted fluorescence was detected by scanned detectors at 490–520 and 575–605 nm and emission filters. Predefined settings for the laser power and detector gain were used for all experiments. Microphotographs were analysed using LAS AF version 2.2.1 (Leica Microsystems) or Volocity 6.01 software.

### Quartz crystal microbalance (QCM)

The interaction of the Naip1 protein with sUA was analysed in a QCM device (Stanford Research Systems (SRS)). A plasmid carrying GFP-tagged Naip1 was virally transduced into THP1 cells, and cell lysates were used to bind the Naip1 protein to anti-GFP previously immobilized onto gold quartz crystals (SRS, 5 MHz). Prior to antibody immobilization, the gold crystals were immersed in piranha solution 1:3H_2_O_2_/H_2_SO_4_ for 15 min, washed twice with absolute ethanol for 5 min, washed three times with ultrapure water for 5 min and dried with a gentle flow of nitrogen. Then, the gold crystals were incubated with EDC (100 mmol/L) and NHS (150 mmol/L), and finally, 200 μL of 20 μg/mL anti-GFP (MA5-15256, Thermo Fisher Scientific) diluted in ultrapure water was deposited over each crystal for 16 h at 4 °C in a humid chamber. The crystals were rinsed in three sequential ultrapure water baths, dried at 22 °C and blocked with 1% BSA solution for 1 h at 37 °C. After incubation, all sample chips were washed and dried as described above. Then, 200 μL of cell lysate was deposited onto the crystals for 16 h at 4 °C in a humid chamber. The crystals were placed in a QCM flow chamber apparatus connected to a syringe pump with a 100 μL/min flow rate (KD Scientific). An initial hydration step with 500 μL of ultrapure water was performed, and 500 μL of each sUA solution sample (12.5, 25, 50, 100 and 200 μΜ) was injected in individual experiments. Each experiment was performed in triplicate. The results are expressed as the average value.

### Surface plasmon resonance (SPR)-based immunosensor development

An Autolab Sprit instrument (Eco Chemie B.V., The Netherlands), which reveals the phenomenon of attenuated total internal reflection (Kretschmann configuration) in operation mode^38^, was employed for the SPR analysis. This SPR instrument was equipped with a glass prism (BK7), a planar gold SPR sensor chip, and two measurement channels (channels 1 and 2). For the measurements, a laser diode with a wavelength fixed at 670 nm and a photodiode detector was employed. In terms of functionality, changes near the metal/environment interface promote a change in the resonance conditions of the system, resulting in a shift in the *θ*_SPR_. In this sense, SPR techniques enable information on biomolecular interactions to be obtained in real time.

The experiments were performed as demonstrated by Souto et al.^39^. Prior to gold surface functionalization, the SPR sensor chip was cleaned in piranha solution (1:3 mixture of 30% H_2_O_2_ to concentrated H_2_SO_4_) for ~1 min, followed by immersion of the substrate in acetone (5 min) and then in isopropyl alcohol (5 min). Next, the SPR sensor chip was washed with deionized water and dried under a pure N_2(g)_ flow. The functionalization of the gold surface was performed through the formation of a self-assembled monolayer (SAM), which was obtained after a 24 h incubation in an ethanol solution consisting of 11-MUA (1.0 mmol/L). After the formation of the film, the gold surface (11-MUA/Au) was copiously washed with ethanol and water and dried under an N_2(g)_ flow. All the steps described above were performed ex situ. In the next step, the functionalized SPR sensor chip was immediately inserted into the SPR instrument, and the measurements were obtained in real time. The terminal carboxyl groups of 11-MUA were activated in a PBS (phosphate-buffered saline) buffer solution (10 mmol/L at pH 7.4) containing EDC (100 mmol/L) and NHS (150 mmol/L) for ~10 min to allow the formation of NHS-ester groups. This strategy was used to allow covalent binding of anti-GFP (20 μg/mL) onto the gold (11-MUA/Au) of the SAM. Next, successive aliquots of the buffer solution were added to remove the excess molecules on the surface. Then, the immobilization of the anti-GFP was monitored via the SPR technique for ~45 min. This step was followed by a washing step using the successive addition of buffer solution. To prevent nonspecific binding, after the immobilization of anti-GFP onto the 11-MUA/Au surface (SAM/Au), the unbound reactive ester groups were deactivated by a PBS buffer solution containing EA (1.0 mol/L at pH 8.5) for ~5 min. Successive additions of the buffer solution then removed the excess unbound EA molecules. After successfully characterizing the immobilization and blocking steps, the interaction between the anti-GFP and Naip1 protein (GFP-tagged) was evaluated. Cell lysates derived from GFP-tagged THP1 cells expressing mNaip1 were used to immobilize Naip1. After characterizing the interaction between anti-GFP and Naip1 protein, an aqueous solution of sUA was added to evaluate the interaction of sUA with Naip1 protein. As a control for this assay, the interaction of palmitate with the Naip1 protein was also evaluated.

### Protein structure analysis

Homology models were obtained using MODELLER v.9.18^40^ with the 4KXF structure as a template^41^. To fix residues with incompatible torsion angles, the target proteins were repaired using RepairPDB^42^, and Chimaera^43^ was used to add hydrogen atoms and charges where appropriate. Surface electrostatic potentials were calculated using the AMBER force field implemented for APBS^44^ and input files that were converted with the PDB2PQR package^45^. For each NAIP structure, blind docking was performed using SwissDock^46^ with UA in its ionized form (urate) as a ligand (ZINC AC: 2041003). During docking, the surface of both proteins was scanned for putative binding pockets with >250 iterations. Several low-energy ligand clusters with similar binding modes (poses) were found. All poses with ∆*G* < −6 kcal/mol were considered in further analyses, and those showing the lowest energies were selected as the best representatives of the binding between urate and both human and mNaip.

### Statistics

Experiments were performed in duplicate or triplicate, and at least two independent tests were performed for each assay. The data are described in terms of the means and s.e.m. unless specified in the figure legend. Differences between groups were compared using analysis of variance (with Tukey’s post-test) and Student’s *t* test. Significant differences were regarded as *p* < 0.05, *p* < 0.01 or *p* < 0.001, according to the figure. All statistical analyses were performed using GraphPad Prism 6.01 (La Jolla, CA, USA). Animals were allocated into groups by similar age and sex. Cells were allocated into groups and treated with plasmids carrying different constructs as specified in the figure legends. In addition, cells were treated with different compounds to trigger or inhibit an inflammasome-mediated response, as stated in the figure legends. No masking or blinding was used for group allocations.

## Results

### Naip1 is involved in the sUA response

**Fig. 1:**
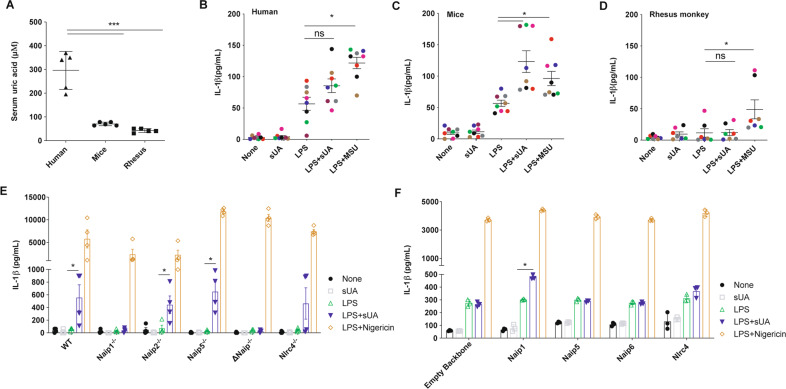
Human cells do not produce IL-1β upon sUA + LPS stimulus, unless they express mNaip1. **A** Uric acid levels measured in the serum of humans, mice and rhesus monkeys. IL-1β Elisa of **B** monocyte-derived macrophages collected from healthy people, **C** murine bone marrow-derived macrophages and **D** monocyte-derived macrophages collected from rhesus monkeys, all stimulated under different conditions. In **B**–**D**, LPS was added for 1 h at 100 ng/mL and the media were posteriorly changed. MSU (100 μg/mL) and sUA (200 μΜ) were added for 6 h. Each coloured dot represents a different individual. **E** IL-1β Elisa of BMDM derived from Naip1^−/−^, Naip2^−/−^, Naip5^−/−^, ΔNaip^−/−^ and Nlrc4^−/−^ mice under different stimulus. **F** IL-1β Elisa of human THP1 cells virally transduced with plasmids carrying Naip1, Naip5, Naip6, Nlrc4 and empty vector using lentivirus constructs and posteriorly stimulated under different conditions. In **E**, **F**, LPS was added for 1 h at 100 ng/mL and the media were posteriorly changed. sUA (200 μΜ) was added for 6 h and nigericin (10 μΜ) was added for 90 min. In **A**, *n* = 5 for each analysed species. Triangle refers to human’s sample, Circle refers to murine’s sample and Square refers to rhesus’ sample. In **B**, **C**, we collected cells from eight different individuals; in **D**, we collected cells from seven different individuals. In **E**, **F**, data are plotted as the median of a triplicate of three to four independent experiments. *p < 0.05 and ***p < 0.001; n.s. not significant.

To assess the difference in serum basal levels of UA among species, we initially measured the UA levels of unrelated healthy adult human donors (*n* = 5), C57Bl/6 mice (*n* = 5), and old-world monkeys (rhesus macaques; *n* = 5). As expected, the human’s samples exhibited an average blood UA concentration of 295 μΜ, 4- and 7-fold greater than the mice and rhesus macaques UA levels, respectively (Fig. 1A). Then, we stimulated murine LPS-primed BMDMs and human and rhesus LPS-primed monocyte-derived macrophages with 200 μΜ sUA. The human cells did not produce IL-1β after sUA stimulus when compared to the level produced by LPS-primed cells (Fig. 1B). On the other hand, murine BMDMs showed increased IL-1β production after sUA stimulation compared to the BMDMs primed with LPS (Fig. 1C). Surprisingly, despite 200 μΜ being supraphysiological for rhesus macaques, the macrophages of these primates did not show increased IL-1β production after the sUA stimulus (Fig. 1D). Ischaemic tissues consistently overproduce UA that triggers immune cell functions^47^. Both sUA stimulation and hypoxic conditions led to increased Naip1 mRNA expression levels in the mouse BMDMs (a 5- and 15-fold increase, respectively) but did not affect Naip5 (Sup. Fig. 1A, B). In addition, BMDMs derived from Naip1^−/−^ and ΔNaip^−/−^ mice did not show increased IL-1β production upon sUA stimulation compared to the amount produced by the LPS-primed macrophages (Fig. 1E). On the other hand, Naip2^−/−^ and Naip5^−/−^ cells behaved as wild-type macrophages, and despite the variation within groups, there were no differences in IL-1β production between the BMDMs from Nlrc4^−/−^ animals after sUA stimulus and the LPS-primed cells from Nlrc4^−/−^ mice (Fig. 1E). To confirm the role of the mNaip platform in the sUA response, we virally transduced human THP1 cells with mNaip1, mNaip5, mNaip6, mNlrc4, and an empty backbone vector. Our data demonstrate that phorbol myristate acetate (PMA)-activated and LPS-primed THP1 cells produced IL-1β after sUA stimulus only after mNaip1 transduction but not mNaip5, mNaip6, mNlrc4 or the control empty vector (Fig. 1F). Such data point to mNaip1 as a target gene involved in the sUA response.

### IL-1β production in human cells is dependent on Nlrp3 and mNaip1

**Fig. 2:**
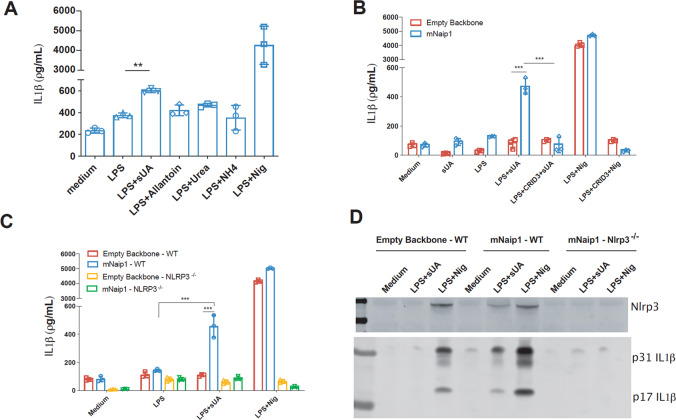
Naip1 and NLRP3 are required for LPS-primed THP1 cells to produce IL-1β upon sUA. **A** IL-1β Elisa of mNaip1-transduced LPS-primed THP1 cells, stimulated with the products of uric acid degradation allantoin, urea and ammonium, the control non-treated cells (Medium) and LPS-primed treated with nigericin. **B** IL-1β Elisa of THP1 cells virally transduced with an empty backbone or with mNaip1 after 1 h pretreatment with LPS (1 µg/mL), 6 h treatment with sUA (200 μM), 30 min treatment with nigericin or control non-treated cells (Medium). Some groups were pretreated with the Nlrp3 inhibitor CRID3 at 1 μΜ 30 min before LPS priming. **C** IL-1β Elisa of WT THP1 and Nlrp3^−/−^ THP1 cells virally transduced with an empty backbone or with mNaip1 after 1 h pretreatment with LPS, 6 h treatment with sUA, 30 min treatment with nigericin or control non-treated cells (Medium). **D** IL-1β and Nlrp3 western blotting images of WT THP1 and Nlrp3^−/−^ THP1 cells virally transduced with an empty backbone or with mNaip1 in control non-treated condition (Medium), or LPS-primed and treated with sUA for 6 h, or with nigericin for 30 min. In **A** blue bars represent the levels of produced IL1beta (pg/mL), analysed by Elisa. In **D**, data are representative of three independent experiments. All experiments were performed three different times and data are plotted as a median of a triplicate. **p < 0.01, and ***p < 0.001.

The Naip1-carrying plasmid was modified by adding a “self-cleaving” 2A (T2A) sequence between the *Naip1* gene and the colour-tagged sequences. In this setup, the overexpressed Naip1 protein is not colour-tagged, which may lead to some data being misinterpreted. Initially, the ability of PMA-activated, LPS-primed and mNaip1-expressing THP1 cells to produce IL-1β upon exposure to UA degradation products, that is, allantoin, urea and ammonium were investigated. None of the products was able to induce IL-1β production, in contrast to the effect of sUA (Fig. 2A). The role of Nlrp3 in sUA sensing, as previously stated^16^, was further investigated. To this end, we initially evaluated IL-1β production upon sUA stimulus of THP1 cells virally transduced with either mNaip1 or the empty backbone stimulated in the presence or absence of an Nlrp3 inhibitor, CRID3 (1 μΜ)^48^. The IL-1β levels were reduced in the cells pretreated with CRID3 (Fig. 2B). IL-1β production was also evaluated in THP1 cells after *Nlrp3* gene deletion by CRISPR-Cas9. It was confirmed that IL-1β production is dependent on Nlrp3 activation (Fig. 2B–D), because Nlrp3-deleted cells exhibited decreased levels of IL-1β upon sUA stimulation (Fig. 2C, D). To investigate the interaction between Naip1 and Nlrp3, immunoprecipitation assays with THP1 cell lysates using both Nlrp3 and Naip1 as targets were performed (Sup. Fig. 2). However, the targets were found only in the whole-cell lysates, not the immunoprecipitates, suggesting that Nlrp3 and Naip1 may not directly interact. Altogether, these data indicate that the observed IL-1β production followed by sUA stimulation requires both Naip1 and Nlrp3 inflammasome platforms.

### Naip1 triggers enhanced immune responses and altered cellular metabolite levels in response to sUA

To clarify the mNaip1 influence on sUA sensing, RNA-Seq analysis of mNaip1- and backbone-transduced THP1 cells was performed. The presence of Naip1 affects gene transcription, which was evident when the gene expression differences are presented in a volcano plot (Sup. Fig. 3A). The unsupervised hierarchical clustering of 8000 genes (Sup. Table 1) and the top 20 most downregulated and most upregulated genes, as determined by the means of the centred logarithm of FPKM (fragments per kilobase of exon model per million reads mapped) values in the six replicates of each experimental group, demonstrated that sUA-stimulated macrophages globally reprogramme transcriptional responses after mNaip1 is expressed (Sup. Fig. 3B). A detailed inspection of the most highly differentially expressed genes revealed that *ccl2*, *pik3cd*, *nck2*, *tab1* and *fgfr1* were expressed more strongly in mNaip1-expressing GFP-tagged cells stimulated with sUA than in GFP-transduced (control) cells stimulated identically (Sup. Fig. 3C). The functional annotation enrichment analyses of KEGG and PANTHER pathways demonstrated that PMA-activated and LPS-primed THP1 cells expressing mNaip1 led to a shift towards increased inflammation-, cancer- and infection-related signalling pathways (Sup. Fig. 3D). The enrichment term analysis using genes that were upregulated in the mNaip1-expressing macrophages revealed a more detailed vision of the KEGG pathway enrichment network (Sup. Fig. 3E). Together, these analyses showed that upregulated genes were associated with processes involved in cytoskeleton regulation, adherent junctions, proteoglycans in cancer, bacterial infection and invasion and the immune response (Sup. Fig. 3E). These data demonstrate that mNaip1 triggers enhanced immune responses after sensing sUA.

In addition to gene expression profiling, we performed a proteomic analysis to identify differentially expressed proteins following sUA stimulation in both control (GFP-transduced) and mNaip1-expressing LPS-primed THP1 cells, and here, we highlight the proteins found only upon exposure to the sUA stimulus in orange and the proteins expressed only under LPS-primed conditions in yellow (Sup. Fig. 4A, Sup. Table 02 and Sup. Table 03). Moreover, the two different cell groups under sUA stimulus were compared (Sup. Fig. 4B). Among the 44 proteins expressed only in mNaip1-expressing cells and not expressed in control cells, we highlighted 30 proteins, including some related to the immune response, such as thymopoietin (A0A024RBH7), CD99 (A8MQT7), stress-associated endoplasmic reticulum protein (Q9Y6X1) and lysosome-associated membrane glycoprotein 2 (H0YCG2) (Sup. Fig. 4B). In addition, the upregulation of mitochondrial citrate synthase (F8VRP1), amino acid transporter (M0R106), mitochondrial glutamate carrier 1 (Q9H936), inorganic pyrophosphatase (Q15181), α-N-acetylgalactosaminidase (P17050), phosphoinositide phospholipase C (B7Z5V4) and acyl-CoA dehydrogenase isoform 4 (A0A0S2Z3A5), which are all associated with cellular metabolism, were observed (Sup. Fig. 4B). We highlight 60 proteins among 342 that were expressed only in the GFP^+^ control cells, including transforming growth factor-beta-induced protein (H0Y8L3), solute carrier family 25-member 4 isoform 3 (A0A0S2Z359) and vimentin (B0YJC4) (Sup. Fig. 4B and Sup. Table 04). Notably, 84% of the analysed proteins assessed in the comparison between the two different cell groups stimulated by sUA were detected in the RNA-Seq data. However, only 2.8% of the expressed proteins corresponded to differentially expressed genes (data not shown). We built a STRING network of only the proteins expressed in mNaip1-expressing cells but not in GFP^+^ control cells, both stimulated by sUA (Sup. Fig. 4C), and the results showed that they were involved in upregulated metabolism-related pathways, especially pathways related to lipid metabolism. These data demonstrate that, in addition to the immune response, mNaip1 is associated with altered cellular metabolite content in LPS-primed macrophages stimulated with sUA.

### Naip1 activation may be potentiated after the elevation of the cellular content of total lipids

**Fig. 3:**
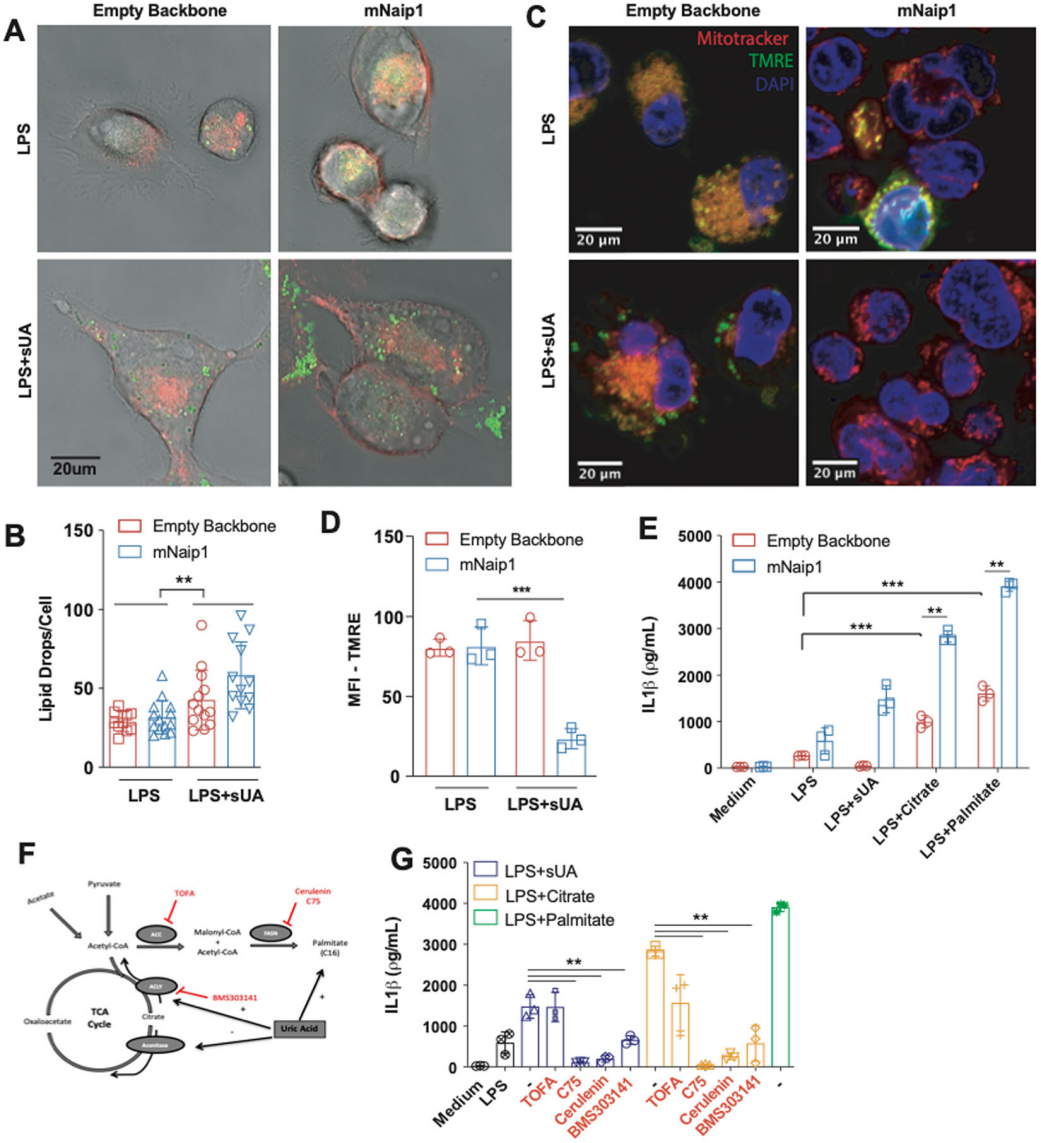
Naip1 activation upon sUA stimulus may be potentiated after the elevation of the cellular content of neutral lipid. **A** Representative images of THP1 cells transduced with an empty backbone or mNaip1 at LPS-primed condition or LPS-primed and stimulated with sUA for 6 h. The membrane is in red and lipid droplets are stained for LD540, in green. **B** Quantification of lipid droplets per cell. **C** Representative images of empty backbone- and mNaip1-transduced cells primed with LPS or LPS-primed and sUA-stimulated stained with MitoTracker (red), tetramethylrhodamine ethyl ester (TMRE) (green) and DAPI (blue). The bars in each image represent 20 μm **D** TMRE quantification, indicating polarized mitochondria of the experiments in panel (**C**). **E** IL-1β Elisa of empty backbone- (red bars) and mNaip1- (blue bars) transduced cells, both at non-stimulated (Medium) condition or LPS-primed and stimulated for 6 h with sUA, citrate or palmitate. **F** Schematic representation of the TCA cycle and the fatty acid synthesis pathway given emphasis to the inhibitors and stimulus used in panel (**J**). **G** IL-1β Elisa of mNaip1-transduced and LPS-primed cells, stimulated for 6 h with sUA (200 μΜ), citrate (5 mΜ) or palmitate (100 μΜ), in the presence or absence of ATP citrate lyase inhibitor (BMS303141 at 25 μΜ), acetyl-CoA carboxylase inhibitor (TOFA at 10 μg/mL), or fatty acid synthase inhibitors (C75 at 50 μΜ or cerulenin at 5 μg/mL). In **A**, **B**, data are representative of three independent experiments and *n* = 12. In **D**, data are plotted as a median of ten different micrography fields of three independent experiments. In **E**, **F**, the experiments were performed three different times and *n* = 3. **p < 0.01 and ***p < 0.001.

UA is also described as increasing the amount of triglycerides accumulated in hepatic cells^49^. Hence, we investigated lipid drop formation in LPS-primed THP1 cells stimulated with sUA. An increase in the cellular content of lipids was observed after sUA stimulation but in a mNaip1-independent manner (Fig. 3A, B). Alterations in metabolite content also lead to changes in mitochondrial activity; once these plastic organelles sense cellular metabolites, oxygen and nutrients, they play central roles as sources of energy and ROS^50^. Changes in mitochondrial membrane potential in live cells upon sUA stimulation were therefore measured (Fig. 3C, D). Despite no differences in mitochondrial area stained with MitoTracker, a reduced mitochondrial membrane potential was observed, as indicated by failure to load the positively charged mitochondrial indicator TMRE in mNaip1-expressing cells stimulated with sUA compared to that in the LPS-primed cells (Fig. 3C, D). We next measured the OCR of THP1 cells virally transduced with an empty backbone or mNaip1, both LPS-primed, treated or not with sUA. sUA increased the OCR but in a Naip1-independent manner. The mitochondrial pyruvate carrier inhibitor UK5099 (100 μΜ) was used to evaluate ATP consumption derived from fatty acids indirectly. Here, the OCR increase was also reversed by UK5099 pretreatment in both cell types (Sup. Fig. 5A, B). Altogether, these data indicate that upon sUA stimulation, mNaip1 expression alters the cellular fatty acid content and reduces the number of active mitochondria in cells.

We next investigated whether the elevation of fatty acid synthesis could trigger IL-1β production. For this assessment, the levels of IL-1β in the supernatant of the LPS-primed cells virally transduced with an empty backbone or mNaip1 were measured after 6 h of incubation with citrate^51^ and palmitate. Despite significant production of IL-1β in the empty backbone-transduced cells upon both citrate (5 mΜ) or palmitate (100 μΜ) stimulation, compared to the LPS-primed cells, the mNaip1-expressing cells produced even higher levels of IL-1β than the empty backbone-transduced cells regardless of the stimuli (Fig. 3E). Moreover, the LPS-primed mNaip1-expressing cells were stimulated with sUA or citrate in the presence of the acetyl-CoA carboxylase-α inhibitor TOFA (10 μg/mL), the phospho-ACLy inhibitor BMS303141^52^ (25 μΜ) or the fatty acid synthase inhibitors C75 (50 μΜ) and cerulenin (5 μg/mL)^53^, as shown in schematic Fig. 3F. All these inhibitors, except TOFA, led to decreased levels of IL-1β (Fig. 3G) upon sUA or citrate stimulation. Altogether, our data suggest that sUA leads to fatty acid synthesis independent of mNaip1 expression. Saturated fatty acids promote Nlrp3 inflammasome activation^54^, especially palmitate^55^. In addition, we found that citrate- and palmitate-mediated IL-1β production is potentiated in the presence of Naip1.

### Naip1 directly recognizes sUA

**Fig. 4:**
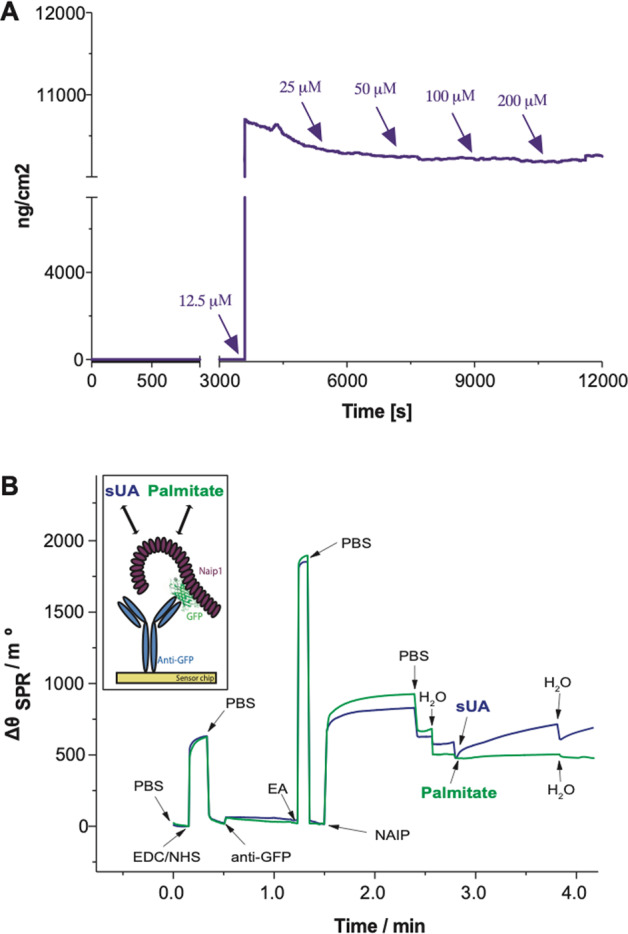
QCM monitoring and SPR sensorgram evidencing all steps involved in the detection of the interaction between sUA and Naip1 protein. **A** QCM responses over time within sUA injection after Naip1 immobilization upon anti-GFP adsorption on the gold quartz crystals surface at 37 °C. The arrows indicate sample injection. **B** Schematic representation of the constructed SPR sensor chip (in the box). Sequential addition of compounds into the system: addition of the buffer solution (PBS, 10 mmol/L at pH 7.4); mixture consisting of EDC (150 mmol/L) and NHS (150 mmol/L); PBS; immobilization of anti-GFP (10 µg/mL); PBS; addition of ethanolamine (EA); addition of cell lysates containing Naip1 protein (2 µg/mol/L). It is possible to observe a very intensive response for the interaction of the Naip1 protein with anti-GFP; PBS; addition of pure H_2_O; addition of sUA (2 µmol/L, purple line) and palmitate (2 µmol/L, green line). It is possible to observe the significant variation of the SPR angle (Δ*θ*_SPR_) due to the interaction between sUA and Naip protein. In **A**, **B**, data are representative of three independent experiments.

In a study investigating the role of different lipids in macrophage lipidomics, palmitate presented the most pronounced effects^56^. To investigate whether mNaip1 directly senses sUA and/or palmitate, we performed a QCM with dissipation (QCM-D) analysis. After an initial immobilizing step with anti-GFP, we incubated the sUA solution sample of increasing concentrations (12.5, 25, 50, 100 and 200 μΜ). It was observed that mNaip1 directly interacts with sUA. As sUA adsorption capacity was reached, the QCM-D frequency changed maximally, even at the lowest studied concentration (Fig. 4A), indicating saturation at lower concentrations and an increase in the mass adsorbed per area. Also, we compared the interaction of mNaip1 stimulated with sUA and with palmitate in real time with an SPR immunosensor. With SPR-based biosensor technology, mNaip1 is tethered to the surface of a previously functionalized SPR sensor chip, and the possible ligands are introduced in solution, as illustrated in the scheme of Fig. 4B. The SPR curves (sensorgram) obtained in real time for all steps involved in the evaluation of the interactions between sUA and mNaip1 protein (purple curve) and between palmitate and the mNaip1 protein (green curve) are shown in Fig. 4B. Notably, a significant variation in response (Δ*θ*_SPR_) was obtained after the addition of sUA (2 µΜ), which characterizes the interaction between sUA and the mNaip1 protein. In this phase of the study, higher concentrations of sUA (12.5–200 µΜ) were also accompanied by significant responses (data not shown). In turn, the addition of palmitate at a concentration of 2 µΜ (green curve) and at higher concentrations (12.5–200 µΜ—data not shown) did not trigger a notable response. These results suggest that sUA directly binds to the mNaip1 protein.

### hNaip and mNaip1 may respond differently to UA

**Fig. 5:**
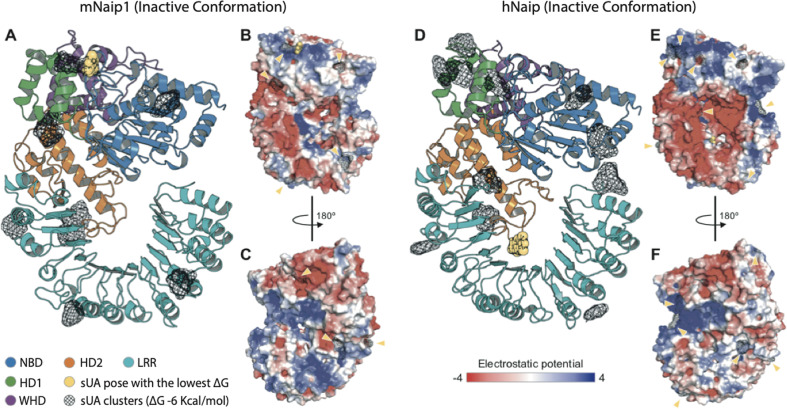
Structural analysis of the inactive conformations of hNaip and mNaip1, modelled by homology using an inactive form of Nlrc4 structure (PDB 4KXF) as a template. **A** Cartoon representation of a mNaip1 (Uniprot: Q9QWK5) homology model. The distinct colours represent functional regions commonly found in proteins of the NLR family (NBD-HD1-WHD-HD2-LRR), coloured as shown in Zhang et al.^57^. Clusters of uric acid (URC) molecules are shown as black meshes, which represent points on the Naip surface where two or more URC were found to bind, in multiple independent rigid docking simulations. The pose with the lowest Δ*G* is shown as a yellow sphere representation. **B** Surface electrostatic potential calculated for mNaip1. Its solvent-accessible surface is shown with a potential gradient ranging from <−4 kBT (red) to >4 kBT (blue). Yellow arrows highlight URC clusters shown in panel (**A**). **C** A 180° rotation of mNaip1 around its *Y*-axis. **D** Cartoon representation of a hNaip (Uniprot: Q13075) homology model. **E** Its surface electrostatic potential. **F** A 180° rotation of hNaip around its *Y*-axis. See legend for more details.

As previously studied, for inflammasomes to be formed, NLR proteins, such as Naip, must recognize the ligands to be released from their autoinhibited state to finally trigger the oligomerization of NLRCs and assemble inflammasome complexes^57,58^. After modelling the structures of mNaip1 and hNaip in their inactive forms, we observed important differences in their surface electrostatic properties, which may directly interfere with their ability to recognize specific ligands. By performing molecular docking, we investigated regions of possible binding of sUA onto the solvent-accessible surface of both Naip proteins (Fig. 5). More than 250 iterations were performed for each target. Given the ligand-binding geometry of each interaction, the docking results were summarized as clusters containing one or more ligands with similar binding poses. Figure 5 displays only clusters with a Gibbs free energy Δ*G* <−6 kcal/mol, with the binding pose with lowest energy (i.e., highest affinity/stability) being highlighted in both targets. As expected, due to their structural differences, the regions where sUA binds in both Naip proteins are mostly discordant. While the predicted binding region of sUA in mNaip1 lies in the NBD domain (Δ*G* = −7.92 kcal/mol) (Fig. 5A), for hNaip, the ligand most likely exhibits higher affinity for the LRR domain (Δ*G* = −8.00 kcal/mol) (Fig. 5D).

Considering our experimental results and the homology modelling and molecular docking, we hypothesize that the surface electrostatics of mNaip1 enables this protein to recognize sUA and stop its autoinhibition, functions that hNaip is unable to perform. With the deletion of the uricase gene in the great apes^19^, throughout evolution, mutations affecting hNaip surface electrostatics were probably selected to increase its physiological tolerance to high levels of sUA such that the activation of inflammasomes is prevented, allowing the great apes to benefit from the survival advantages provided by high levels of serum UA^21^.

## Discussion

Among all mNaip proteins, Naip1 presents the highest amino acid content similarity with hNaip, ~70%. It is possible that the 30% difference between human and murine proteins is associated with sUA signalling. In addition, it has been demonstrated that Naip/Nlrc4 inflammasome activation is sufficient to cause systemic inflammatory disease with surprising tibiotarsal joint swelling^59^, the main joint affected by gout, a disease triggered by the accumulation of UA^60^. This is the first study to postulate that Naip recognizes a DAMP. Our previous work suggested that sUA activates the Nlrp3 inflammasome^16^; hence, we also investigated the role of Nlrp3 in mNaip1-expressing cells. LPS-primed human THP1 cells produce only the mature form of IL-1β upon sUA stimulation when they also express Nlrp3 and mNaip1. Most inflammasomes are believed to include only a single NLR, although other NLR-NLR interactions have been proposed. The interplay between Nlrp3 and Nlrc4 reveals an unexpected overlap between inflammasome scaffolds previously thought to be distinct^61^. In addition, it was reported that Nlrc4 can recruit Nlrp3 through its NACHT domain in the context of *Salmonella typhimurium* infection^61^. Hence, in addition to the mechanisms by which sUA activates the mNaip1 inflammasome, it remains to be determined whether mNaip1 interacts with Nlrp3 after sUA stimulation, as we observed no clear evidence of Nlrp3 and mNaip1 protein interactions.

In addition to responding to PAMPs and DAMPs, the immune system acts as a signal integrator able to detect disturbances in the cytoplasm related to metabolites, as indicated by recent data. These monitored disruptions are termed “homeostasis-altering molecular processes”^62^. It has been shown that Nlrp3 inflammasome complex activation and posterior caspase-1 and IL-1β production occur following saturated fatty acid palmitate triggering, even in human cells^63,64^. Both QCM and SPR methodologies indicate that the three-dimensional structure of mNaip1 provides a great accessible area for interaction with sUA and no accessible area for interaction with palmitate. Homology modelling and molecular docking analysis indicate that the surface electrostatics of mNaip1 enable it to recognize sUA by abolishing its autoinhibition state, a function that hNaip is unable to perform. Although we have demonstrated that sUA, but not palmitate, is responsible by directly binds to mNaip1, our data pointed to increased IL-1β production in the context of Naip1 expression in human cells following palmitate stimulation. Therefore, further analyses are necessary to better investigate the mechanisms by which palmitate can lead to Naip1 permissiveness to sUA.

In addition, the transcriptome and cellular metabolite contents were changed in cells upon sUA stimulation, and the presence of mNaip1 altered some metabolism-related enzymes and favoured an increase in immune responses towards sUA. Moreover, we observed that mitochondrial activity was inhibited in mNaip1-expressing cells stimulated with sUA. This result corroborates the findings of a study demonstrating that the percentage of TMRE^+^ cells was significantly lower in LPS-primed macrophages stimulated with ATP than in control macrophages^65^.

Uricase activity that is missing because of evolutionary process endows UA a puzzling history in the evolution of humans. Great apes have, in the basal state, high physiological levels of sUA, but human macrophages do not respond to 200 μΜ sUA, an inflammatory-inducing stimulus in murine cells. Uricase inhibition therapy and the subsequent elevation in serum UA are critical for triggering metabolic syndrome comorbidities in murine models^66–68^. We have demonstrated, on the other hand, that rhesus macaques, a primate with evolutionarily maintained uricase enzyme activity, have reduced levels of IL-1β production by their monocyte-derived macrophages following sUA stimulation. It is possible that rhesus macaques’ macrophages require higher priming activation to induce IL-1β transcription since LPS alone did not increase IL-1β production in these experiments. Moreover, several cytokines, including IL-1β, circulate at very low levels in both affected and unaffected rhesus macaques in different models of disease^69–71^. It is also speculated that the rhesus Naip protein was selected because it tolerates elevated levels of serum UA.

In recent years, an understanding of the additional adverse effects of high levels of serum UA has advanced^72^. Early scientific literature suggested an association between UA concentration and the incidence of cardiovascular disease; specifically, the development of hypertension^73^, metabolic syndrome^74^, endothelial dysfunction^75^ and microalbuminuria^76^. Lifestyle and socioeconomic changes over time have resulted in a marked reduction in physical activity and profound dietary changes. These changes correlate with increased rates of metabolic diseases triggered by overly active innate immune functions^77^, with chronic inflammation termed “metaflammation”^78,79^. Furthermore, multiple genetic and non-genetic risk and protective factors are also thought to contribute to the pathogenesis of metabolic diseases, specifically those related to hyperuricaemic conditions. Different states of tolerance to sUA sensing by hNaip may predict the innate immune activation state in the context of hyperuricaemia-related diseases. These tolerance differences may be related to polymorphisms in the *Naip* gene, changes in the number of gene copies, or even epigenetic changes; however, a greater investigation is required. In this sense, in addition to understanding the human evolutionary process, investigating which mechanisms mediate the immunomodulatory function of sUA is also essential to better design rational novel anti-inflammatory therapies.

## Supplementary information

Supplemental Figure and Table Legends

Sup Fig. 01

Sup Fig. 02

Sup Fig. 03

Sup Fig. 04

Sup Fig. 05

Sup. Table 01

Sup. Table 02

Sup. Table 03

Sup. Table 04
