# Supplementary material for: Sensing soluble uric acid by Naip1-Nlrp3 platform
Source: Cell Death Dis. 2021 Feb 5;12(2):158. doi: 10.1038/s41419-021-03445-w (PMC7864962; doi:10.1038/s41419-021-03445-w)
Supplement: Supplementary file 1 — Supplemental Figure and Table Legends [file 41419_2021_3445_MOESM1_ESM.docx]

**Supplemental Figure and Table Legends**

**Supplemental Figure 01.** **Soluble uric acid induces Naip1, but not Naip5, mRNA expression.** Naip1(black bars) and Naip5 (gray bars) mRNA levels in bone marrow-derived macrophages after (**A**) sUA stimulus for 24 hours into LPS-primed cells when compared to non-treated cells, and (**B**) hypoxia condition for 24 hours, when compared to normoxia condition. qPCR data were normalized to HPRT expression, and the mean expression in (A) non-treated cells and (B) normoxia condition was considered 1. n = 3 animals per group in each experiment. **p < 0.01, ***p < 0.001.

**Supplemental Figure 02.** **Nlrp3 and Naip1 may not directly interact to each other upon sUA stimulus.** Immunoprecipitation - western blot analysis on THP1 whole cell lysates (WCL) and on THP1 immunoprecipitants (IP) using Nlrp3 mAb (upper panel) and Naip1 polyclonal Ab (lower panel) followed by anti-Naip1 and anti-Nlrp3 antibodies, respectively. Cells were analyzed under three different conditions: non-stimulated (medium), LPS-primed and sUA-stimulated (LPS+UA), and LPS-primed and nigericin-stimulated (LPS+Nig). Figures are representative of three different experiments.

**Supplemental Figure 03.** **THP1 cells transduced with mNaip1 shows increased expression of inflammation-related genes after sUA treatment.** THP1 cells were transduced with a lentiviral vector containing NAIP1 and GFP (NAIP1) and only with GFP gene (control). Cells were sorted on the basis of their GFP fluorescence followed by LPS pretreatment and sUA treatment then mRNA was purified and sequenced. (**A**) Volcano plot of gene expression changes. The y-axis specifies the negative logarithm to base 10 of t-test *P-*values and x-axis specifies the logarithm of fold changes to base 2. Colored dots indicate significantly differential expressed genes (q-value < 0.05). Vertical lines reflect threshold criteria for up-regulated genes (log_2_ fold change > +0.5) colored in blue (n=49), down-regulated genes (log_2_ fold change < -0.5) colored in red (n=24), and not differentially expressed genes in light grey (n=175). (**B**) Top 20 most down and up-regulated genes in NAIP1 cells (q-value < 0.05) by means of centered logarithm of FPKM values in the six replicates of each experimental group. (**C**) Box-plot showing FPKM expression levels (q-value < 0.05) of selected genes *CCL2* (NM_002982), *PIK3CD* (NM_005026), *NCK2* (NM_003581), *TAB1* (NM_006116), and *FGFR1* (NM_023106) in control and NAIP-1 cells. (**D**) Heatmap of enriched KEGG 2016 and Panther 2016 database terms as columns and differentially expressed (q-value < 0.05 and n=248) genes as rows. Enriched terms (*P*-value < 0.05) were grouped as inflammation, infection or cancer-related major groups. (**E**) Network of enriched terms on KEGG database found in enrichment analysis of only up-regulated genes (q-value < 0.05, fold change > 1, and n=136). Each node represents a KEEG 2016 term and links represents that a term (node) have some genes in common. KEEG terms are highlighted in inflammation, infection and cancer major groups. The experiments were performed in duplicates in three independent analysis.

Proteomics

analysis

in

AT

showing

upregulated

proteins

in

DIO

WT

versus

lean

WT

(STRING

netwo

rk

view:

colored

lines

between

the

proteins

indicate

the

various

types

of

inte

raction

evidence).

Proteomics

analysis

in

AT

showing

upregulated

proteins

in

DIO

WT

versus

lean

WT

(STRING

netwo

rk

view:

colored

lines

between

the

proteins

indicate

the

various

types

of

inte

raction

evidence).

Proteomics

analysis

in

AT

showing

upregulated

proteins

in

DIO

WT

versus

lean

WT

(STRING

netwo

rk

view:

colored

lines

between

the

proteins

indicate

the

various

types

of

inte

raction

evidence)

Proteomics

analysis

in

AT

showing

upregulated

proteins

in

DIO

WT

versus

lean

WT

(STRING

netwo

rk

view:

colored

lines

between

the

proteins

indicate

the

various

types

of

inte

raction

evidence)

**Supplemental Figure 04.** **sUA triggers altered cellular protein content in cells expressing mNaip1.** (**A**) Proteomic analysis of LPS-primed or LPS-primed and sUA-stimulated macrophages transduced with empty backbone (left panel) or mNaip1 (right panel) showing proteins only present in each condition. The numbers indicate the amount of proteins exclusively expressed in each condition. (**B**) Proteomic analysis of LPS-primed and sUA-stimulated macrophages showing thirty proteins only present in mNaip1-expressed cells (in the yellow circle) and sixty proteins only present in empty backbone-transduced cells (in the orange circle). The numbers indicate the amount of proteins exclusively expressed in each condition. (**C**) STRING network view of proteomics analysis showing upregulated proteins in mNaip1-expressing cells versus empty backbone-transduced THP-1 ones, both after sUA stimulus. Colored lines between the proteins indicate the various type of interaction evidence. The experiments were performed in triplicates.

**Supplemental Figure 05.** **sUA triggers increased maximal respiration in a mNaip1-independet way.** (**A**) Bioenergetic profiles of THP-1 cells under different stimuli. Cells (60,000 per well) were treated with respiratory inhibitors and uncoupler at the following concentrations: oligomycin (1 μg/mL), CCCP (5 μΜ) and antimycin A (10 μg/mL) plus rotenone (1 μΜ). The graph shows representative oxygen consumption rates (OCR) from LPS-primed control cells (light red line), LPS-primed and sUA-stimulated control cells (red line), LPS-primed and sUA-stimulated control cells under UK5099 (100 μΜ) pre-treatment (dark red line), LPS-primed Naip1 expressing cells (light blue line), LPS-primed and sUA-stimulated Naip1 expressing cells (blue line), and LPS-primed and sUA-stimulated Naip1 expressing cells under UK5099 pre-treatment (purple line). (**B**) Area under curve of graph in A. Data are representative of three independent experiments and n = 7 for each analyzed condition. *p < 0.05.

**Supplemental Table 01.** The unsupervised hierarchical clustering of 8,000 genes determined by the means of the centred logarithm of FPKM values in the six replicates of each experimental group: GFP-control or Naip1 overexpressed cells under control stimulus (LPS-primed cells) or LPS-primed and sUA-stimulated ones.

**Supplemental Table 02.** Proteomic analysis demonstrating differentially expressed proteins following sUA stimulation in **mNaip1-expressing** LPS-primed THP1 cells, in the comparison between cells not stimulated with sUA. Proteins found only upon exposure to the sUA stimulus are highlighted in orange and the proteins expressed only under LPS-primed conditions, in yellow.

**Supplemental Table 03.** Proteomic analysis demonstrating differentially expressed proteins following sUA stimulation in **control (GFP-transduced)** LPS-primed THP1 cells, in the comparison between cells not stimulated with sUA. Proteins found only upon exposure to the sUA stimulus are highlighted in orange and the proteins expressed only under LPS-primed conditions, in yellow.

**Supplemental Table 04.** Proteomic analysis demonstrating 547 differentially expressed proteins following sUA stimulation in **both** **control (GFP-transduced) and mNaip1-expressing** LPS-primed THP1 cells. Proteins found in control cells are highlighted in orange and the proteins expressed in mNaip1 expressing cells, in yellow.
