# Supplementary figures and images for: Sensing soluble uric acid by Naip1-Nlrp3 platform
Source: Cell Death Dis. 2021 Feb 5;12(2):158. doi: 10.1038/s41419-021-03445-w (PMC7864962; doi:10.1038/s41419-021-03445-w)

### LPS+sUA

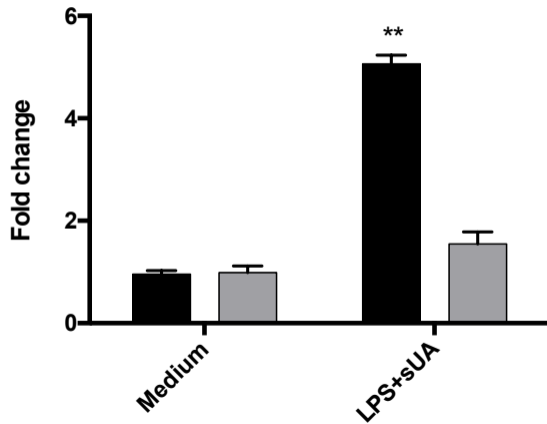

### Hypoxia

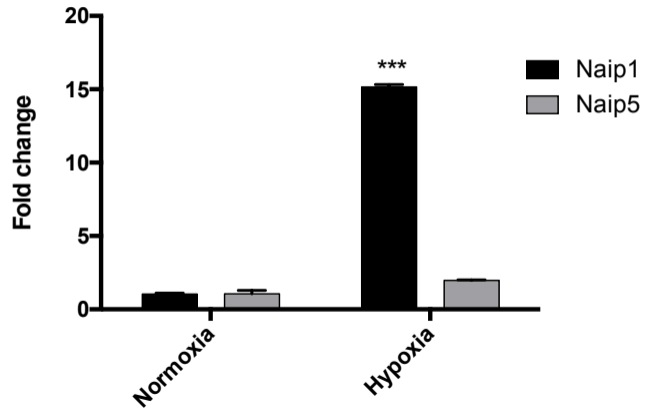

Supplement: Supplementary file 2 — Sup Fig. 01 [file 41419_2021_3445_MOESM2_ESM.pdf]

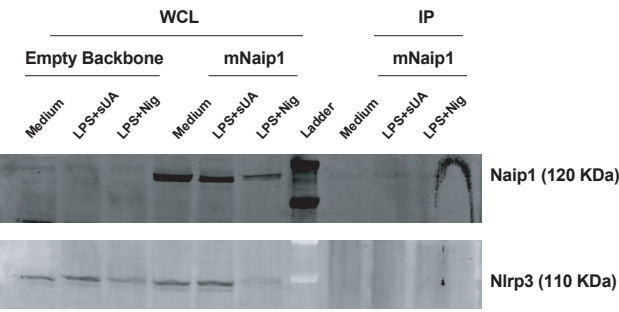

Supplement: Supplementary file 3 — Sup Fig. 02 [file 41419_2021_3445_MOESM3_ESM.pdf]

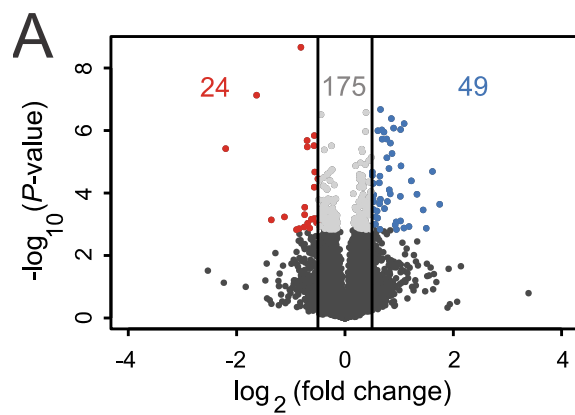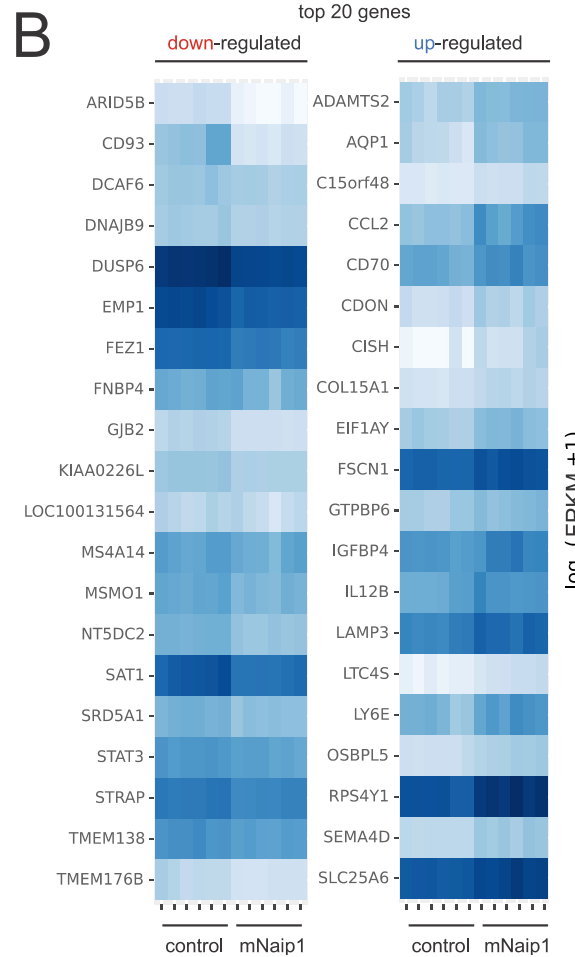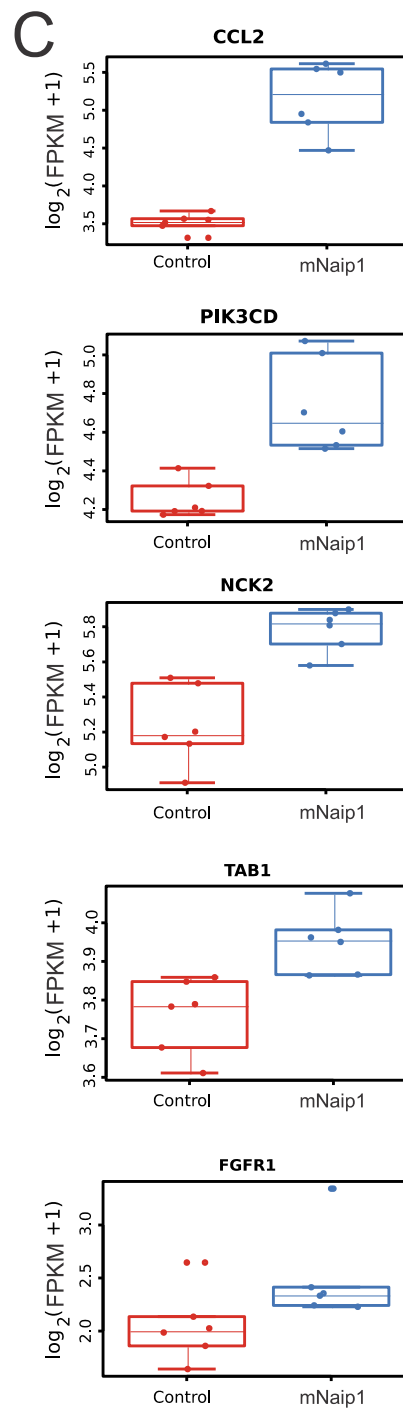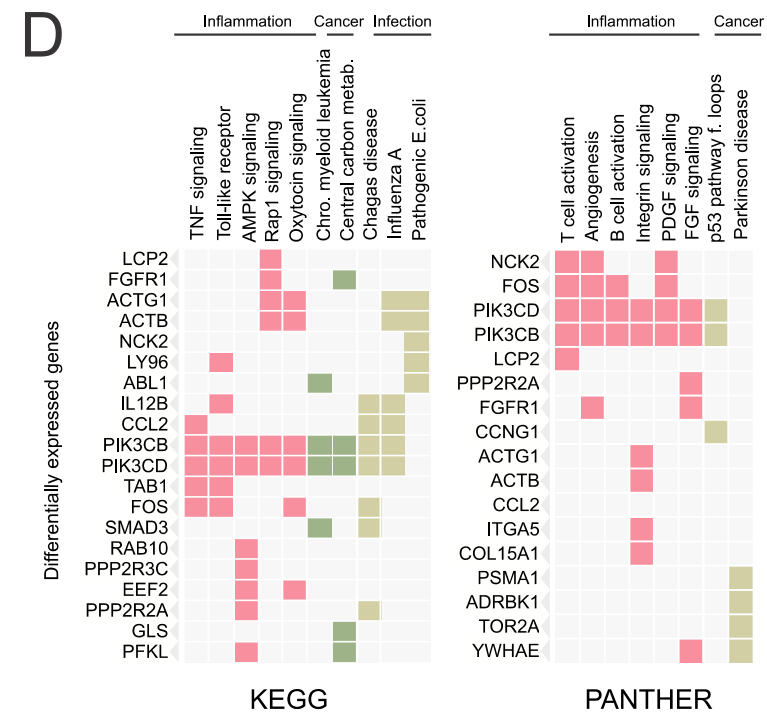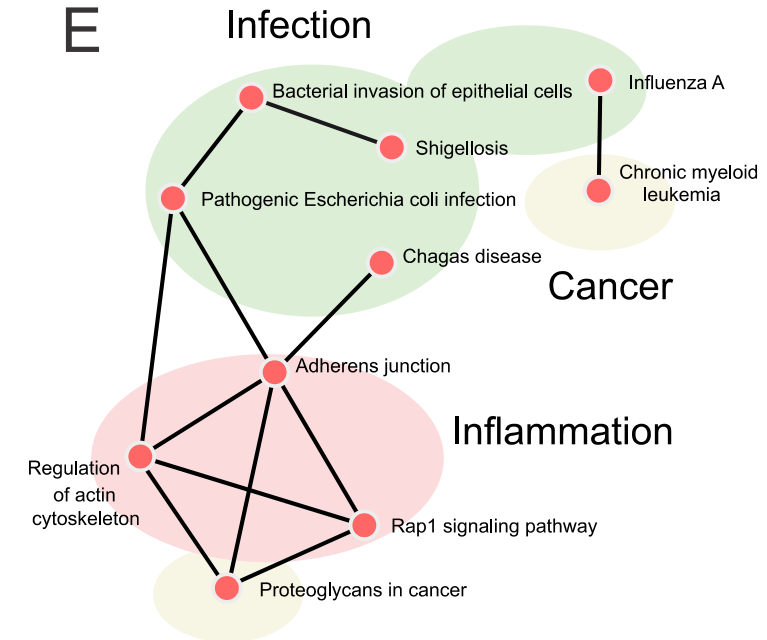

Supplement: Supplementary file 4 — Sup Fig. 03 [file 41419_2021_3445_MOESM4_ESM.pdf]

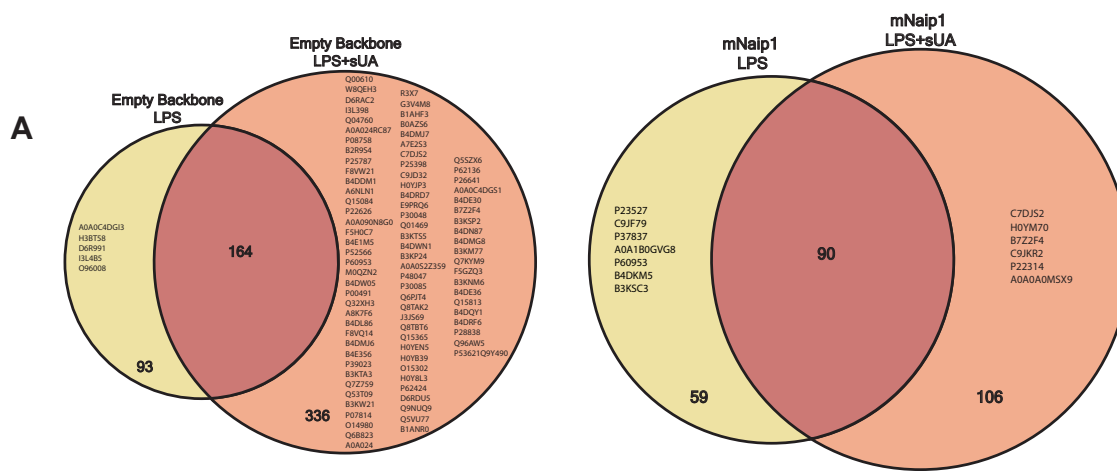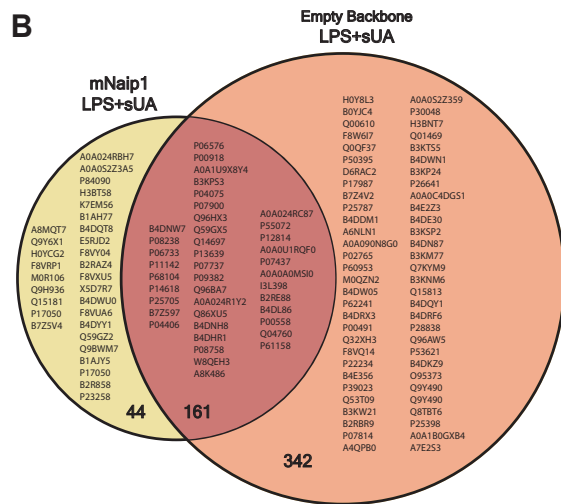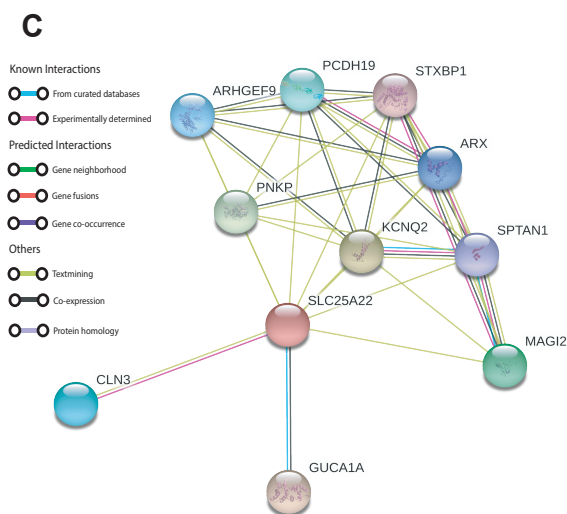

Supplement: Supplementary file 5 — Sup Fig. 04 [file 41419_2021_3445_MOESM5_ESM.pdf]

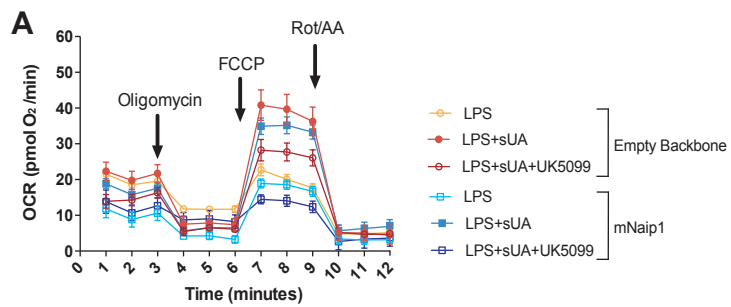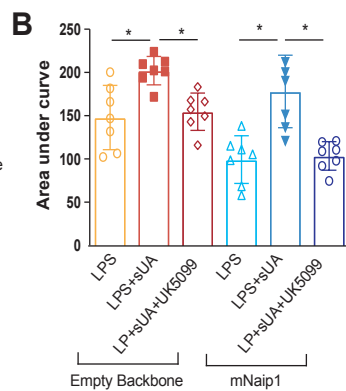

Supplement: Supplementary file 6 — Sup Fig. 05 [file 41419_2021_3445_MOESM6_ESM.pdf]
